# Assessment of Serum Urea, Creatinine and Uric Acid in Oral Cancer

**DOI:** 10.3390/jcm11123459

**Published:** 2022-06-16

**Authors:** Ana Caruntu, Liliana Moraru, Diana Alina Ciubotaru, Cristiana Tanase, Cristian Scheau, Constantin Caruntu

**Affiliations:** 1 Department of Oral and Maxillofacial Surgery, “Carol Davila” Central Military Emergency Hospital, 010825 Bucharest, Romania; ana.caruntu@gmail.com (A.C.); liliana.moraru@yahoo.com (L.M.); diana-alina.ciubotaru@drd.umfcd.ro (D.A.C.); 2 Department of Oral and Maxillofacial Surgery, Faculty of Dental Medicine, “Titu Maiorescu” University, 031593 Bucharest, Romania; 3 Proteomics Department, Cajal Institute, Faculty of Medicine, “Titu Maiorescu” University, 031593 Bucharest, Romania; cristianatp@yahoo.com; 4 Department of Biochemistry-Proteomics, “Victor Babes” National Institute of Pathology, 050096 Bucharest, Romania; 5 Department of Physiology, “Carol Davila” University of Medicine and Pharmacy, 050474 Bucharest, Romania; costin.caruntu@gmail.com; 6 Department of Dermatology, “Prof. N.C. Paulescu” National Institute of Diabetes, Nutrition and Metabolic Diseases, 011233 Bucharest, Romania

**Keywords:** urea, creatinine, uric acid, oral cancer, metabolism, squamous cell carcinoma

## Abstract

Background: Oral squamous cell carcinoma (OSCC) is a common malignancy worldwide, leading to significant disease-associated social and financial burdens. The investigation of underlying mechanisms involved in carcinogenesis and tumor progression in OSCC might provide new therapeutic perspectives with an impact on disease control and patient survival. Our study aims to investigate the interrelation between metabolic processes, expressed through final catabolism products and clinicopathological characteristics in OSCC. Materials and methods: This is a single cancer comparative retrospective study investigating metabolic byproducts, namely serum urea, creatinine and uric acid, detected at the moment of diagnosis in patients with OSCC, in comparison to healthy controls. Clinical and paraclinical data regarding exposure to risk factors, disease staging and pathological characteristics were collected for all patients. Subjects with co-existing systemic or metabolic diseases, or with a history of malignancy, were excluded from the study. Subsequently, the metabolic byproducts revealing significant changes in OSCC patients were considered for a correlation analysis with the disease clinico-pathological characteristics. Results: Blood levels for urea, creatinine and uric acid were determined in a total of 225 subjects: 145 patients diagnosed with OSCC and 80 healthy control subjects admitted to our hospital between 2016 and 2021. The comparative analysis between groups revealed that the serum urea level was significantly lower in OSCC patients (*p* = 0.0344). Serum creatinine and uric acid did not reveal significant differences between groups. Furthermore, in advanced stages of the disease (stages III and IV), the blood level of urea was significantly lower compared to incipient OSCC (stages I and II) (*p* = 0.003). We found a negative correlation of serum urea levels with smoking (*p* = 0.0004) and cervical lymph node metastasis (*p* = 0.0070), and a positive correlation with aging (*p* = 0.0000). We found no significant correlation of serum urea with primary tumor size (*p* = 0.5061) and patient survival (*p* = 0.2932). Conclusions: Decreased serum urea levels are detected in patients with advanced OSCC, in correlation with lymph node metastasis. The invasive features of tumor cells in OSCC might be promoted in association with dysregulation of protein catabolism processes, facilitating aggressive behavior in OSCC.

## 1. Introduction

Oral squamous cell carcinoma (OSCC) is a common malignancy worldwide, with an incidence of more than 350,000 new cases per year [1]. The paradox about this malignancy is related to the simplicity of its diagnosis; on one hand, clinical suspicion usually being raised after a routine oral examination, and the high incidence of advanced disease found at the first presentation on the other hand. In more than half of the cases, OSCC is diagnosed in late stages, with locally advanced or metastatic disease found at the moment of first presentation. This leads to dramatic consequences in terms of survival rates, despite all the progress achieved by modern therapeutic strategies [2,3]. In addition, the quality of life of these patients is significantly affected due to post-treatment sequelae, often severely mutilating, with functional impairment, and having a major social impact on the patients [4,5]. Furthermore, the economic burden associated with oral cancer is significant in countries with high incidence [6]. Considering all these aspects, great efforts are being invested to unveil the underlying mechanisms of carcinogenesis, tumor growth and invasion in OSCC. In many cancer types, recent scientific discoveries translated into novel therapeutic strategies have revolutionized the medical world, turning cancer into a curable disease [7]. However, despite all progress made in surgical and non-surgical treatment strategies in OSCC, survival rates have not improved significantly during the last decades, except when the disease was diagnosed and treated in early stages [8,9]. Recent scientific literature provides important results in the investigation of different pathogenetic mechanisms in oral cancer, such as the involvement of genetic, immune or inflammatory elements in cancer emergence and progression [10,11,12,13], leading to novel approaches in terms of disease characterization and treatment strategies [14,15,16,17].

In recent years, metabolic changes and their relation to cancer pathogenesis have raised the interest of the scientific community in the quest for efficient “weapons” to fight against cancer. The increased demand for energy in cancer cells, required to support their specific malignant features, is secured through genetically controlled, complex reprogramming of different metabolic pathways [18]. Some of these mechanisms have been studied and alterations in specific metabolic pathways, such as aerobic glycolysis or fatty acid oxidation, are currently known to be key players in tumor growth and invasion [19]. In addition, dysregulation of anabolic and catabolic processes has been reported in many types of cancer [20,21]. These findings were linked with carcinogenesis and tumor growth [18,22]. In this context, metabolism byproducts, such as serum urea, uric acid or creatinine, classically used for monitoring liver and kidney function, might enclose important information regarding cancer pathogenesis [23,24]. Recent studies link intense purine catabolism, expressed through elevated serum uric acid, the final metabolite of purine degradation, with higher incidences of cancer and cancer-associated deaths [25]. Similarly, contrasting changes in serum urea levels, the final metabolite of amino acid degradation, were reported in hepatocellular and renal carcinomas in females, suggesting that this parameter may be used as an early detection alternative for these types of malignancies [26,27]. Creatinine, the final product of arginine and glycine degradation, has also been investigated in different types of cancer, sustaining its prognostic character in several types of epithelial cancers [28,29,30]. However, in oral cancer, scientific data regarding the assessment of protein metabolism processes and their specific pathways is scarce, sustaining the need for an in-depth investigation to clarify the complex metabolic events associated with oral cancer pathogenesis.

Our study aims to investigate the interrelation between the systemic metabolic processes expressed in the serum biochemistry profile and the clinicopathological characteristics in OSCC. For this purpose, we considered the evaluation of several end catabolism products, routinely determined in a clinical setting in OSCC patients, namely serum urea, creatinine and uric acid.

## 2. Materials and Methods

### 2.1. Patients’ Samples

We have included patients diagnosed with OSCC, admitted to the Department of Oral and Maxillofacial surgery, of the “Carol Davila” Emergency University Military Hospital Bucharest, between January 2016 and December 2021. Primary tumor sites were the tongue, floor of the mouth, buccal mucosa, gingiva, palate mucosa and lips. All patients underwent an initial general assessment, represented by systemic and local clinical examination, blood tests and imaging evaluation. Patients with preexisting or newly diagnosed systemic diseases associated with increased creatinine, urea or uric acid levels, such as renal or hepatic failure, or gout, were excluded from the study. Similarly, patients with other decompensated metabolic or systemic diseases or patients with a history of other types of malignancies were also excluded from the study. Based on the clinical examination and imaging criteria, clinical TNM staging of the disease was determined. Patients underwent treatment according to national guidelines. Radical tumor resection was conducted in all resectable lesions. Ipsilateral or bilateral neck dissection (when primary tumor crossed the midline) was performed in all cases of suspected lymph node metastasis. Prophylactic neck dissection was conducted in primary tumors with increased risk for nodal spread, including large oral tumors exhibiting an ulcerative growth pattern, located in the posterior oral cavity. Adjuvant treatment, represented by radiotherapy with or without chemotherapy, was indicated according to the loco-regional spread of the disease, and determined after pathology assessment of the specimen. All patients were included in a follow-up program, consisting of periodical clinical examinations and imaging.

In the control group, we have included subjects admitted in the same department between 2019 and 2021 for minor oral surgery procedures (teeth extractions, small benign oral lesion removal), with no history of associated systemic disease and no abnormal values of their blood tests. Demographic criteria—age and gender distribution—were comparable between the two study groups.

The study was conducted with the approval of the Local Ethics Committee from our hospital (no 484/2021).

### 2.2. Data Collection

Clinical and laboratory data for all patients included in our study were collected from patients’ charts and the electronic database of our hospital. At admission, as part of the pre-treatment workup, all patients underwent a complete screening program, represented by general and local clinical examination, imaging assessments, cardiac and respiratory examinations, and complete blood tests, plus additional investigations for individual cases when needed. Among laboratory markers, the final products of the metabolism—serum urea, creatinine and uric acid (elements of interest in our study)—were determined. Only pre-treatment samples were considered for the analysis.

### 2.3. Statistical Analysis

We have conducted the statistical analysis with SPSS software version 23 (IBM). Kolmogorov–Smirnov and Shapiro–Wilk tests were used to assess the normality of data distribution within the groups. Subsequently, data with parametric distribution was analyzed using the Independent Student T-test, whereas data with non-parametric distribution was analyzed using the Mann–Whitney U test. Comparative analysis between multiple groups was performed with the Independent Samples Kruskal–Wallis Test followed by the post hoc test of Pairwise Comparison for each pair of groups. Biological parameters demonstrating significant differences in OSCC compared to healthy controls were selected for correlation analysis with clinicopathological characteristics of OSCC, using the bivariate Spearman model. Multiple regression analysis using the backwards stepwise method was subsequently conducted. Data were reported as mean values plus/minus standard deviation (SD) or median and interquartile range (IQR) for the relevant biological parameters depending on the model of data distribution. Statistical significance was considered to be *p* < 0.05.

## 3. Results

### 3.1. Patients’ Group Characteristics

From a total of 197 patients admitted to our hospital with a diagnosis of OSCC, 145 met the eligibility criteria and were included in our study. The other 52 patients were excluded based on the above-mentioned criteria: preexisting decompensated conditions or insufficient clinical data. The general characteristics of our study group are presented in Table 1. The mean age in our OSCC group was 63.32 years old (ranging from 33–92), with an important predominance of males, who represented 79% of the patients. A total of 97 patients were smokers, whereas alcohol abuse was confirmed in almost half of the patients. Patient underwent radical surgery consisting of tumor excision with immediate defect reconstruction through primary closure, local, regional or distant flaps. In 121 patients, ipsilateral or bilateral neck dissection was performed depending on the midline involvement by the primary tumor. Disease staging revealed that more than half of the patients (57%) were diagnosed in advanced stages of the disease. Locally extended tumors, exceeding 4 cm or invading adjacent anatomical structures, staged T3 and T4, were found in 56 patients (39%). Regional spread in the cervical lymph nodes was confirmed in 61 patients (42%). The rest of the 60 patients submitted to neck dissection had negative lymph node involvement after the pathology assessment of the specimen. Histologically, more than half of the tumors were classified as moderately differentiated (55%), 21% were poorly differentiated, and the rest were well-differentiated. The mean follow-up time in our study group was 36.40 months, ranging between 2 and 80 months. During the follow-up period, 30 (21%) patients died secondarily to disease progression, whereas 115 were alive at the last follow-up visit.

In the control group, 80 healthy subjects were included—62 males (78%) and 18 females (22%) with a mean age of 61.6 ± 13.02, ranging between 29 and 85 years old. The two groups were similar in terms of age and gender characteristics, with no statistically significant differences (*p* = 0.3159 for age and *p* = 0.8469 for gender).

### 3.2. Comparison of Serum Urea, Uric Acid and Creatinine Levels between OSCC and Control Groups

Comparative analysis between the OSCC group and healthy subjects revealed significant differences in serum urea. Thus, in OSCC patients, serum urea levels were significantly lower compared to the control group (*p* = 0.0344), with a mean value of 33.07 mg/dl in the OSCC group compared to a mean value of 35.30 mg/dl in the control group. For the other two biological parameters—creatinine and uric acid—we found no significant differences between our groups. The results of our analysis are enclosed in Table 2.

### 3.3. Analysis of Serum Urea in Relation to Disease Progression in OSCC

**Figure 1:**
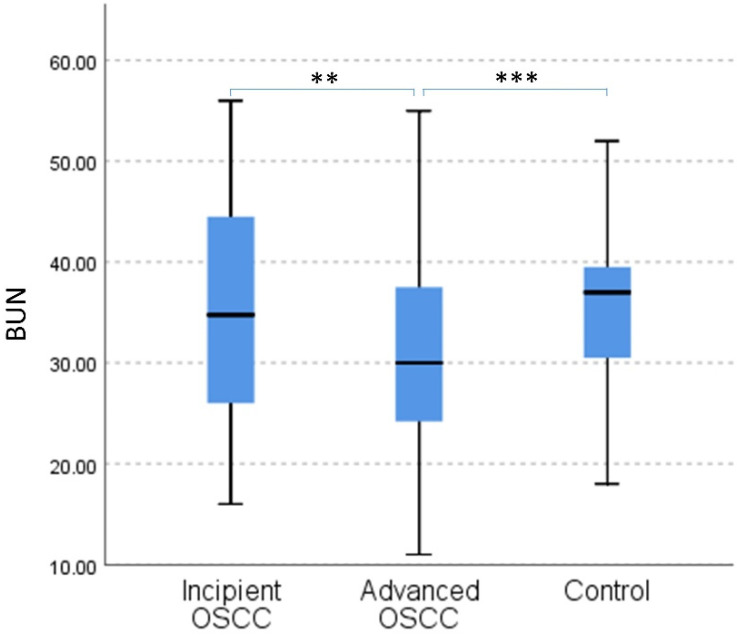
Independent Samples Kruskal–Wallis Test pairwise comparison of blood urea nitrogen (BUN) in the three groups. OSCC = Oral squamous cell carcinoma; ** *p* ≤ 0.01; *** *p* ≤ 0.001.

Considering our initial findings, we have focused on serum urea for our further analysis. We conducted a comparative analysis of serum urea in relation to disease progression and against the control group using the Independent Sample Kruskal–Wallis test, which revealed significant differences between the three groups (*p* = 0.001) (Figure 1). The pairwise assessment revealed that in incipient OSCC, corresponding to limited local disease with no regional spread, there were no differences between serum urea levels compared to healthy subjects (*p* = 0.969). However, when compared to advanced disease, defined as locally or regionally spread OSCC, we found a significant decrease in serum urea levels in those with advanced disease compared to incipient OSCC (*p* = 0.003). Furthermore, the trend was similar in the comparison between healthy subjects and patients with advanced disease. Thus, significantly lower serum urea levels were found in the Advanced OSCC group compared to the control group (*p* = 0.001). The mean value for serum urea in patients with advanced OSCC was 30.91 (±9.03) mg/dl, compared to a mean value of 35.96 (±10.76) mg/dl in incipient disease and 35.30 (±7.04) mg/dl in the control group.

### 3.4. Correlation and Multivariate Analysis between Serum Urea and Clinico-Pathological Characteristics in OSCC

Considering these results, we conducted a correlation analysis between serum urea in OSCC and other clinico-pathological characteristics of the disease including survival. Our findings revealed a positive correlation between serum urea and age in OSCC (r = 0.409, *p* < 0.000). In addition, we found significant correlations of serum urea levels with smoking status and cervical lymph node metastasis. Thus, in smoking OSCC patients, there was a negative correlation with serum urea levels (r = −0.300, *p* = 0.0004). Similarly, in patients with confirmed lymph node invasion, we found a negative correlation with serum urea levels (r = −0.244, *p* = 0.007). Contrary to our expectations, considering the difficulties associated with food intake in large oral tumors, in our study group, we did not find a significant correlation between the primary tumor size and serum urea levels (r = −0.056, *p* = 0.5061). In OSCC, for other characteristics such as alcohol consumption, histological differentiation and patients’ survival, we did not find any significant correlations with serum urea levels. The mean value of serum urea for each category of OSCC, as well as correlation analysis results, are presented in Table 3.

## 4. Discussion

Protein catabolism results in the release of disposable nitrogen molecules, amongst others, under the form of ammonia, a highly toxic metabolite [31]. In normally functioning organisms, detection of ammonia activates specific pathways to dispose of this excess metabolite. Thus, ammonia is converted to urea, a hydrosoluble, non-toxic metabolite excreted through urine. This process takes place mainly in the liver and is known as the urea cycle, the first metabolic cycle described in the medical scientific literature [32]. A complete urea cycle transforms two molecules of nitrogen into one molecule of urea and involves five catalytic enzymes plus two membrane transporters [33]. Recent studies suggest a potential link between this metabolic pathway, specifically dysregulation of the urea cycle, and cancer pathogenesis [34]. All body cells are equipped with at least some of the urea cycle enzymes, as these are tightly linked with other metabolic processes, such as the tricarboxylic acid cycle, and in many cells, specific urea cycle enzymes are the sole source of several endogenous amino acids [35]. In cancer cells, urea cycle dysregulation promotes cell proliferation by redirecting nitrogen molecules, from the normal process of disposal in the form of urea to anabolic processes resulting in molecule biosynthesis, thus facilitating cancer growth [36]. Experimental models on cell lines for ovarian cancer, hepatocellular carcinoma and melanoma have confirmed that perturbations of urea cycle enzymes enhance malignant cells proliferation, through a detour of the nitrogen substrate toward pyrimidine biosynthesis, using the path of CAD activation (Carbamoyl-phosphate synthetase 2, Aspartate transcarbamylase and Dihydrooratase) [23]. In breast cancer cell lines, excess ammonia was not processed into urea but was incorporated into amino acids, and subsequently used for the biosynthesis of macromolecules, lipids and nucleotides, providing a source of energy to the metabolically depleted cells [20]. Furthermore, in lung cancer cell lines, increased activity of mitochondrial urea cycle enzymes was detected. However, it did not lead to an elevation of urea discharge but instead led to the incorporation of metabolic intermediate carbamoyl phosphate for the synthesis of pyrimidine molecules [21]. In the clinical setting, altered activity of specific urea cycle enzymes was reported in colorectal carcinoma with a significant negative impact on patient survival and response to therapy [37].

In our investigation, based on clinical findings in patients diagnosed with OSCC, serum urea levels were significantly lower compared to healthy controls. Furthermore, we found a continuous decrease in serum urea levels in association with disease progression to advanced stages. Recent studies conducted on animal models and oncologic patients report similar findings in other types of malignancies. Decreased serum levels of urea were detected in mice with colon cancer, together with reduced expression of urea cycle enzymes, suggesting a deviation from the normal metabolism of nitrogen to urea towards the anabolic process of pyrimidine biosynthesis [23]. The same authors report results from a retrospective study involving pediatric cancer patients, in which significantly lower serum urea levels were detected on admission day compared to age-matched healthy controls [23]. Another study comparing saliva metabolites from patients with OSCC and healthy controls reported significant differences in 25 out of 499 metabolites between the two groups, including urea. More intriguing was the fact that all the metabolites revealed higher values in OSCC compared to controls except urea, for which significantly lower saliva values were found in OSCC patients [38].

Age-related changes in serum urea levels were confirmed in our study groups, with increasing serum urea levels reported in correlation with aging, supporting the homogeneity of our groups. Extensive studies of healthy subjects presented a progressive elevation of blood urea with aging, with significant differences being detected with every decade of aging in both genders [39,40,41]. We also have found a significant negative correlation between serum urea levels and smoking in our study group. These results are in accordance with previous reports, that identified important differences in blood urea levels in smoking subjects, who exhibited decreased serum urea values compared to non-smokers [42]. The underlying mechanisms associated with these changes are still unknown, but a direct link can be suspected, considering that significant differences were detected for some of the urea cycle intermediate metabolites, such as aspartate, in smokers versus non-smokers [43].

It is known that serum urea is influenced by protein intake [44]. In consequence, we suspected a potential decrease in serum urea levels in our OSCC patients in the context of a poor dietary regimen, caused by the presence of large tumors in the oral cavity which are usually associated with functional disability. However, the results of our analysis did not confirm this hypothesis. We found no significant correlation between the dimensions of the primary tumor and serum urea levels in our group of patients. Conversely, significant correlations were confirmed between the decrease of serum urea levels and the regional spread of the disease into the cervical lymph nodes in OSCC. Another study conducted on patients with head and neck cancers, assessing the predictive character of different pretreatment laboratory parameters, reports similar findings regarding blood urea levels [45]. Two-thirds of the patients included in the study were in advanced stages of the disease and low serum urea levels were detected in these patients that did not carry a prognostic value but were significantly correlated with lymph node metastasis. Contrasting with our findings, the authors also reported significantly lower urea levels in patients with large tumors, staged T3 and T4. Impaired eating and swallowing, commonly encountered in large head and neck tumors, could explain changes in metabolic byproducts detected in body fluids [46,47]. However, similar findings reported in other types of malignancies sustain more complex underlying mechanisms, probably through metabolic shifting towards the synthesis of necessary elements for the highly demanding energetic needs specific to neoplastic cells [19]. Recent studies have suggested a link between urea cycle alterations and increased metastatic potential in cancer cells, attributed to modifications within the tumor microenvironment that impair the local immune response and facilitate tumor cell migration in regional or distant sites [34]. In head and neck squamous cell carcinoma, changes in the expression of urea cycle enzymes in the tumor microenvironment, specifically arginase II, were correlated with tumor immune infiltration with T regulatory cells and CD11+ myeloid dendritic cells [48]. Studies on prostate and ovarian carcinomas report similar findings, revealing an altered immune response correlated with changes in the expression of urea cycle enzymes [49,50]. But the impaired local immune response is not the only element that facilitates cancer cell metastasis. The plasticity of epithelial cancer cells, also known as epithelial-mesenchymal transition, provides the specific features to allow cancer cell migration to regional or distant sites [51]. It has been shown that deprivation of asparagine, the biosynthesis product of asparagine synthetase, led to a decreased metastatic behavior in cancers [52]. Ammonia and aspartate, both reagents of the urea cycle, are the main substrate for asparagine biosynthesis [53], suggesting that a deviation of these molecules, could be expressed clinically in metastatic cancers in decreased serum urea levels.

However, the most important aspect in unveiling urea cycle dysregulation associated with cancer pathogenesis is the therapeutic potential that might be developed, to enhance the arsenal of anticancer strategies, especially in malignancies that did not show major improvements in disease control with currently available treatments. A multitude of molecules, including vaccines, that target different urea cycle components—enzymes or intermediate metabolites—are currently under investigation, alone or in combinations, and preliminary results show promising anticancer effects [54,55,56].

Our investigation reports the clinical expression of metabolic changes associated with nitrogen disposal mechanisms in OSCC, raising the attention for the need for an in-depth analysis of the underlying pathways which might influence disease progression and spread in this type of malignancy. To our knowledge, this is the first report suggesting an interrelation between serum urea changes and cancer progression in OSCC. Interestingly, our results are concordant with the above-mentioned study on head and neck cancer patients, where the pharynx and larynx sites were predominant compared to oral primary tumors [45], thus supporting our findings. The limitations of our study are related to the single-center character and the relatively limited number of subjects. In addition, a complete overview of the protein intake in our patients could provide a more accurate perspective on the subsequent metabolic changes in OSCC. Our results are a starting point for future investigations in the field of metabolic alterations and OSCC pathogenesis, using in vivo or in vitro experimental models, with the purpose to unveil the underlying mechanisms which might lead to these changes and opening the path for novel therapeutic strategies against cancer.

## 5. Conclusions

OSCC pathogenesis is far from being unveiled. Decreased serum urea levels are detected in advanced stages of the disease, in association with lymph node metastasis, which might suggest an interrelation between the dysregulation of protein catabolic processes and the aggressive behavior of cancer cells in OSCC. Considering the complex context of genetic, immune and systemic alterations that contribute to tumor progression and distant spread, the need for an in-depth investigation of the interconnections with metabolic changes is obvious in order to provide a more comprehensive picture of cancer pathogenesis in OSCC.

## Figures and Tables

**Table 1:** OSCC group characteristics.

Total Patients (145)		No.	%
Age	Mean ± SD	63.32 ± 11.83 (ranging 33–92)	
Gender	Males	114	79
	Females	31	21
Smoking status	Yes	97	67
	No	40	28
	Missing data	8	6
Alcohol abuse	Yes	70	48
	No	67	46
	Missing data	8	6
TNM staging	1	19	13
	2	43	30
	3	28	19
	4	55	38
Disease status	Incipient (TNM 1–2)	62	43
	Advanced (TNM 3–4)	83	57
Primary tumor dimensions	Small (T stages 1–2)	89	61
	Large (T stages 3–4)	56	39
Lymph node metastasis *	Positive neck	61	42
	Negative neck	60	41
Histological differentiation degree	Well-differentiated	35	24
	Moderately differentiated	80	55
	Poorly differentiated	30	21
Disease related survival	Alive	115	79
	Deceased secondarily to disease progression	30	21

* neck dissection performed for 121 patients.

**Table 2:** Comparison of serum urea, uric acid and creatinine between OSCC group and Control group.

	Serum Urea		Creatinine		Uric Acid	
	OSCC Group	Control Group	OSCC Group	Control Group	OSCC Group	Control Group
No subjects	145	80	145	80	116 #	72
Mean/Median (mg/dL)	32.00	33.00	0.7600	0.7750	5.267	5.338
SD/IQR	15.55	10.25	0.2350	0.2125	1.361	1.193
*p* value	**0.0344** ^		0.2090 ^		0.7190 *	

# missing uric acid data for 29 patients in OSCC group; * independent student *t*-test; ^ Mann–Whitney U test; SD—standard deviation; IQR—interquartile range; statistical significance < 0.05 (bold characters).

**Table 3:** Correlation analysis between serum urea and clinic-pathological characteristics in OSCC patients.

Parameter		Mean	SD	Correl. Coeff.	***p*** **Value**
Age				**0.409**	**0.0000**
Gender	Males	32.67	10.14	0.075	0.3714
	Females	34.52	9.932		
Smoking	Smokers	30.98	9.689	**−0.300**	**0.0004**
	Non-smokers	37.35	9.016		
Alcohol abuse	Confirmed	31.19	9.083	−0.156	0.0679
	No alcohol abuse	34.56	10.48		
T stage	Small tumors (T1–2)	33.49	10.4	−0.056	0.5061
	Large tumors (T3–4)	32.39	9.628		
Lymph node invasion *	Positive nodes	30.24	8.797	**−0.244**	**0.0070**
	Negative nodes	35.16	10.63		
Histological differentiation degree	Well-differentiated	34.03	9.738	−0.035	0.6793
	Moderately differentiated	32.65	10.28		
	Poorly differentiated	33.06	10.23		
Disease related prognosis	Alive	33.51	9.771	−0.088	0.2932
	Deceased secondarily to disease	31.36	11.24		

* Neck dissection performed for 121 subjects; Spearman test, statistical significance < 0.05 (bold characters). The multiple regression model using backwards stepwise method confirmed age, smoking and lymph node involvement as predictors for changes in serum urea level in OSCC (*p* = 0.001, *p* = 0.019 and *p* = 0.031 respectively). Results are presented in Table 4.

**Table 4:** Multiple regression analysis for serum urea in OSCC patients.

Model		β	Std. Error	*p* Value
1	Age	**0.295**	**0.086**	**0.001**
	Gender	−1.742	2.339	0.458
	Smoking status	**−4.624**	**2.238**	**0.041**
	Alcohol abuse	−0.549	2.122	0.796
	Histological differentiation	0.171	1.311	0.897
	T stage	0.166	0.893	0.853
	Lymph node invasion	**−1.831**	**0.910**	**0.047**
	Disease related prognosis	0.287	2.153	0.894
2	Age	**0.288**	**0.082**	**0.001**
	Smoking status	**−4.510**	**1.888**	**0.019**
	Lymph node invasion	**−1.702**	**0.779**	**0.031**
Dependent variable: serum urea				

Bold characters represent statistical significance < 0.05.

## Data Availability

The data presented in this study are available on reasonable request from the corresponding author.
